# Supplementary material for: Catheter Ablation for Atrial Fibrillation in Patients with Heart Failure with Preserved Ejection Fraction: A Systematic Review and Meta-Analysis
Source: J Clin Med. 2022 Jan 6;11(2):288. doi: 10.3390/jcm11020288 (PMC8779551; doi:10.3390/jcm11020288)
Supplement: Supplementary file 1 [file jcm-11-00288-s001.zip › jcm-1339030-supplementary.pdf]

Table S1. Quality Assessment Scale for Case Series Studies – National Heart, Lung, and Blood Institute [15].

**Criteria**	**Studies**										
	**Cha et al (2011)**	**Machino-Ohtsuka et al (2013)**	**Black-Maier et al (2018)**	**Ichijo et al (2018)**	**Saksena et al (2018)**	**Kelly et al (2019)**	**Vecchio et al (2019)**	**Machino-Ohtsuka et al (2019)**	**Eitel et al (2019**	**Fukui et al (2020)**	**Elkaryoni et al (2020)**
1. Was the study question or objective clearly stated?	Yes	Yes	Yes	Yes	Yes	Yes	Yes	Yes	Yes	Yes	Yes
2. Was the study population clearly and fully described, including a case definition?	Yes	Yes	Yes	No	Yes	No	Yes	No	Yes	No	No
3. Were the cases consecutive?	Yes	Yes	Yes	Yes	Yes	Yes	Yes	Yes	Yes	Yes	Yes
4. Were the subjects comparable?	No	No	No	No	No	No	No	No	No	No	No
5. Was the intervention clearly described?	Yes	Yes	Yes	Yes	Yes	Yes	Yes	Yes	No	Yes	Yes
6. Were the outcome measures clearly defined, valid, reliable, and implemented consistently across all study participants?	Yes	Yes	Yes	No	Yes	Yes	Yes	Yes	Yes	Yes	Yes
7. Was the length of follow-up adequate?	Yes	Yes	Yes	Yes	Yes	Yes	Yes	Yes	Yes	Yes	Yes
8. Were the statistical methods well-described?	Yes	Yes	Yes	No	Yes	Yes	Yes	Yes	Yes	No	Yes
9. Were the results well-described?	Yes	Yes	Yes	Yes	Yes	Yes	Yes	Yes	Yes	Yes	Yes
